# Artificial Intelligence and Ophthalmology

**DOI:** 10.4274/tjo.galenos.2020.78989

**Published:** 2020-03-05

**Authors:** Kadircan Keskinbora, Fatih Güven

**Affiliations:** 1 Bahçeşehir University Faculty of Medicine, Department of Ophthalmology, Division of Medical Ethics and History of Medicine, İstanbul, Turkey; 2 Health Sciences University Bakırköy Training and Research Hospital, Clinic of Ophthalmology, İstanbul, Turkey

**Keywords:** Artificial intelligence, machine learning, deep learning, ophthalmology, medical ethics

## Abstract

Artificial intelligence is advancing rapidly and making its way into all areas of our lives. This review discusses developments and potential practices regarding the use of artificial intelligence in the field of ophthalmology, and the related topic of medical ethics. Various artificial intelligence applications related to the diagnosis of eye diseases were researched in books, journals, search engines, print and social media. Resources were cross-checked to verify the information. Artificial intelligence algorithms, some of which were approved by the US Food and Drug Administration, have been adopted in the field of ophthalmology, especially in diagnostic studies. Studies are being conducted that prove that artificial intelligence algorithms can be used in the field of ophthalmology, especially in diabetic retinopathy, age-related macular degeneration, and retinopathy of prematurity. Some of these algorithms have come to the approval stage. The current point in artificial intelligence studies shows that this technology has advanced considerably and shows promise for future work. It is believed that artificial intelligence applications will be effective in identifying patients with preventable vision loss and directing them to physicians, especially in developing countries where there are fewer trained professionals and physicians are difficult to reach. When we consider the possibility that some future artificial intelligence systems may be candidates for moral/ethical status, certain ethical issues arise. Questions about moral/ethical status are important in some areas of applied ethics. Although it is accepted that current intelligence systems do not have moral/ethical status, it has yet to be determined what the exact the characteristics that confer moral/ethical status are or will be.

## What is Artificial Intelligence?

Artificial intelligence, described simply, is the ability of a computer to mimic the intellectual intelligence unique to humans. This type of intelligence includes qualities such as the ability to link events to specific causes, make generalizations, and learn from experience.^1^ As a general public notion, the term is used to describe devices that can provide a reason for a certain phenomenon, develop strategies, make judgments about situations, and have the ability to learn. However, there are ongoing controversies regarding the level and reliability of this intelligence.^2^

Many different theories on how to evaluate machine intelligence have been proposed. The most famous of these is the Turing test, which was put forward in 1950 by Alan Turing, an English mathematician, computer scientist, and cryptologist. In this test, an assessor compares responses given by a computer and a person without knowing who gave which answer and predicts which one is the machine. If the machine can convince the assessor with its answers at least 30% of the time, it passes the test. In 2014, a program called Eugene Goostman passed this test.^3^

### Types of Artificial Intelligence

Artificial intelligence is classified under three headings based on technological achievements and future projections:

**1) Artificial Narrow Intelligence:** Artificial narrow intelligence, which encompasses nearly all of the software currently described as artificial intelligence, mimics human intelligence within the limited field for which it is designed and responds within this framework.

**2) Artificial General Intelligence:** This type of artificial intelligence has the same intellectual capacity as humans and is expected in theory to be able to perform tasks at the same level as a person. The consensus among researchers of artificial intelligence is that this type, also called human-level artificial intelligence, must be able to learn and reason, develop strategies, make plans, communicate using language, and synthesize all of these abilities to accomplish a certain task.^4^

**3) Artificial Superintelligence:** This kind of artificial intelligence is expected to be superior to the most intelligent and talented human brain, and prominent figures in science and technology such as Stephen Hawking and Elon Musk have suggested grim scenarios for the future related to its emergence.

### Artificial Intelligence Learning Algorithms and Ophthalmology

**a) Machine Learning:** The term “machine learning”, one of the subclasses of artificial intelligence frequently used in ophthalmology studies, was first introduced in 1959 by the engineer Arthur Samuel, a pioneer in artificial intelligence. He defined this term as the ability of machines to learn outcomes that are not explicitly programmed.^5^

In the machine learning technique, the aim is to generate an algorithm based on a certain amount of data entered into a computer and for the computer to then use this algorithm to improve its predictions. The phase in which the device trains with the input to improve its predictions is the learning phase, which is divided into two types: supervised and unsupervised learning. In supervised learning, labels are assigned to the training data as they are entered into the computer, while in unsupervised learning, the device creates its own algorithm from unlabeled input.

**b) Deep Learning: **As the machine learning technique improves and the amount of input increases, this more advanced method uses multiple layers to generate output, unlike machine learning, which operates with a single layer. Using deep neural networks, the computer can train with much larger data capacity and improve itself with each training cycle to create its own algorith.

### Examples of Artificial Intelligence in Medicine

As in many other industries, the use of artificial intelligence in the field of medicine is steadily increasing. Major companies in numerous medical sectors, particularly the pharmaceutical and imaging sectors, have invested billions of dollars in this field, while research on artificial intelligence software is also an area of intense interest in the academic sphere. Although the various publications on the use of artificial intelligence applications in different fields of medicine reveal the breadth of the uses of these techniques, the number of studies that have been approved is still limited.

To give some notable examples of artificial intelligence applications in the field of medicine, in 2016 an artificial intelligence framework by Google called DeepVariant was proven to be able to identify single nucleotide polymorphisms, the most common genetic variation, with 99.9587% accuracy and received an award from the FDA.^6^ The OsteoDetect application, used for wrist fractures in adults, evaluates patients’ X-ray images and was approved by the FDA in 2018.^7^

Artificial intelligence applications developed for purposes such as diagnosing tuberculosis by evaluating a chest X-rays, assessing suspected malignant melanoma based on skin lesion photographs, and detecting lymph node metastasis of breast cancer by analyzing pathology slides, and publications about these represent examples of future areas of use of artificial intelligence.^8,9,10,11^ This is exemplified by a radiology algorithm developed at Stanford University that was able to diagnose pneumonia more accurately than radiologists.^12^

### What Can Artificial Intelligence Do for Ophthalmology?

The field of ophthalmology is well suited for artificial intelligence studies, with its numerous digital techniques such as color fundus photography, optical coherence tomography (OCT), and computerized visual field (VF) testing and the huge databases they provide. 

In addition to this, the global increase in life expectancy is accompanied by an increase in eye diseases that cause preventable vision loss.^13,14^ Solutions are sought for the early diagnosis and treatment of these diseases, especially in regions where access to physicians is difficult. Artificial intelligence applications are being developed for many different eye diseases, particularly diabetic retinopathy (DR), age-related macular degeneration (AMD), glaucoma, and retinopathy of prematurity (ROP), which are the leading causes of vision loss.^15^

### Artificial Intelligence and Diabetic Retinopathy

Due to the rapidly increasing number of patients worldwide, DR has generated the most interest in terms of the use of artificial intelligence in ophthalmology. IDx-DR, the first FDA-approved device using artificial intelligence software, was also developed for this area.^16^

The IDx-DR uses a Topcon NW400 fundus camera to classify patients according to retinopathy level. Ease of use was cited as the priority when choosing the fundus camera. The operators selected to obtain the fundus photographs had no previous experience using a fundus camera. Patients were grouped into those with mild to advanced DR according to the American Academy of Ophthalmology classification (Preferred Practice Patterns for Diabetic Retinopathy) and those without retinopathy, and the patients were recommended follow-up examination at 12 months or sooner according to their results. A total of 900 patients participated in the study and the sensitivity and specificity of the device were found to be 87.4% and 89.5%, respectively. The device began to be used at the University of Iowa in 2018 after receiving FDA approval.^16^

The IDx-DR was developed using software that uses deep learning techniques, and there are a growing number of similar studies using fundus cameras and deep learning software.^17,18,19,20^ Thanks to deep learning applications, it is possible to develop software with databases containing over 100,000 data points.^21,22^

There are examples of studies using machine learning methods with fundus photographs, machine learning with OCT, and deep learning methods with OCT.^19,23,24,25,26,27^ Some of these studies have reported nearly 100% sensitivity or specificity rates.^28,29^

### Artificial Intelligence and Age-related Macular Degeneration

As with DR, an increasing number of studies are investigating software that uses artificial intelligence for the early diagnosis and classification of AMD. The earliest published studies involved software developed using fundus photography and machine learning with database sizes smaller than 1,000.^30,31,32^ Later, with software using deep learning technology, database sizes increased and high sensitivity and specificity rates were reached.^20,33,34^

Ting et al.^20^ used a database of 72,610 fundus photographs and classified patients as those with intermediate to advanced AMD and those without according to the AREDS (Age-Related Eye Disease Study) classification. They reported sensitivity and specificity of 93.2% and 88.2%, respectively.^20^

Burlina et al.^33^ classified patients with software developed using 130,000 images from 4613 patients and reporting a 91.6% accuracy rate in identifying those with moderate and advanced AMD patients.

Grassmann et al.^34^ tested an algorithm they generated from 120,656 fundus photographs of 3,654 patients against the AREDS database and reported an accuracy rate of 84.2% in differentiating early and late disease and 94.3% accuracy in identifying healthy subjects.

### Artificial Intelligence and Glaucoma

Glaucoma is among the leading causes of vision loss worldwide and has also attracted the attention of artificial intelligence researchers due to the importance of its early diagnosis and treatment.^15^

Initially, studies using machine learning to identify glaucomatous optic nerve damage based on fundus photographs were published.^35,36,37^ These were followed by studies that used deep learning technology with much larger databases compared to the earlier machine learning studies.^20,38,39^ In another study using a database of 125,189 fundus photographs, Ting et al.^20^ reported a sensitivity of 96.4% and specificity of 87.2%.

Studies are being conducted on the use of imaging modalities other than fundus photography in the diagnosis and monitoring of glaucoma. In addition to artificial intelligence applications created using computerized VF and OCT data, studies have also been published describing programs that are able to evaluate patients based on data from both of these examination devices.^40,41,42,43,44,45^

### Artificial Intelligence and Retinopathy of Prematurity

ROP is a leading cause of vision loss in childhood worldwide and its prevalence is reported as 6-18% in different studies.^46^ According to the ETROP (Early Treatment for Retinopathy of Prematurity) study, early treatment is vital for improving visual acuity, and 9% of patients have permanent vision loss despite early treatment.^47^

Although the impact of ROP diagnosis and treatment on patients’ visual acuity outcomes and quality of life is known, access to physicians specializing in ROP can be limited, especially in less developed countries. One of the reasons for this is that the follow-up and treatment of ROP requires a long time and specialized education, even for ophthalmologists. This coupled with high malpractice rates and lawsuits result in physicians avoiding this area.^48,49^ In addition, parameters used in the diagnosis of ROP, such as zone, stage, and presence of additional diseases lead to diagnostic variations even among ROP specialists.^48^ The difficulty in finding specialists and the diagnostic variations among specialists has prompted artificial intelligence researchers to conduct studies on ROP.

Brown et al.^50^ developed software using deep learning technology and a database of 5,511 fundus images obtained with a RetCam fundus camera and reported 93% sensitivity and 94% specificity in determining the presence of additional disease.

In an application developed by Redd et al.^51^ based on the same deep learning technology, the software was found to have 0.96 and 0.91 area under the curve values, respectively, in the identification of type 1 ROP and clinically significant ROP.

### Other Applications in Ophthalmology

Data from a study by De Fauw et al.^52^ conducted at Moorfields Eye Hospital using Google’s deep learning technology called DeepMind presents a striking illustration of the level artificial intelligence has reached.

The artificial intelligence algorithm was trained by introducing 10 different lesions such as hemorrhage and fluid and using 14,884 untagged OCT images, and is able to distinguish more than 50 retinal diseases. The study included data obtained from 37 different OCT devices from different centers affiliated with Moorfields, and data from the application were compared with the decisions of four ophthalmologists and four optometrists affiliated with Moorfields. These physicians and the device were asked to classify OCT images based on the need for referral as urgent, semi-urgent, routine examination, and observation.

At the end of the study, the software’s error rate (5.5%) was comparable to those of the hospital’s two best retina specialists (6.7% and 6.8%) and was significantly better than those the other six specialists (10-24.1%). In particular, it was reported that the software made no errors in the urgent referral group.^52^

## Discussion

The progress made in artificial intelligence studies shows that important advances in this technology are ongoing, and it is clear that potential future applications lie on the horizon and are promising for future studies. It is believed that artificial intelligence will be effective in identifying patients with preventable vision loss and referring them to a physician, especially in developing countries where access to physicians is difficult and the population of trained individuals is low.

The diagnostic spectrum in which artificial intelligence may be used and its possible clinical benefits represent a broad field of study. With technologies similar to those in previous studies, applications based on different imaging modalities can be developed in various areas such as occlusive vascular disease, keratoconus, and retinitis pigmentosa. Beyond just screening for or diagnosing diseases, surgical applications may be created to provide guidance for physicians in areas such as determining the ideal type of intraocular lens for a patient or estimating the risk of surgery. 

Another important aspect of artificial intelligence that should be discussed is the potential ethical conflicts. Imagine that in the near future, a bank evaluates home loan applications using machine learning algorithms. Now imagine that someone denied a loan sues the bank, claiming that the algorithm racially discriminated in its evaluation. The bank will state that this is impossible because the algorithm does not know the applicants’ race. In fact, the reason the bank implemented such a system was to eliminate unpleasant situations such as the involvement of human emotions. Nevertheless, suppose statistics indicate that the bank’s approval rate is steadily falling for black applicants. When ten equivalent applications are entered into the system, the algorithm accepts those of the white applicants and rejects those of the black applicants. What do you think is happening here? It may not be easy to determine the answer. If the machine learning algorithm is created using a complex artificial neural network or based on a genetic algorithm generated by controlled evolution, it would be impossible to understand why and according to what data the algorithm makes its racially discriminant decisions. In contrast, decision trees or networks in special computer language are much more transparent in terms of allowing the programmer to analyze them. This may allow an auditor to discover, for example, that the decision is reached based on where the applicants were born or the fact that they previously resided mostly in suburban neighborhoods, i.e., their addresses.

Artificial intelligence algorithms play an increasingly prominent role in modern society. In general, however, those who are affected by them are not even aware that such a thing exists in the background.

When we consider the possibility that some future artificial intelligence systems may be candidates for moral/ethical status, various ethical issues arise. Relations with beings of moral/ethical status are not entirely a matter of rationality; we also have moral/ethical reasons to treat them in certain ways and to avoid mistreating them. Kamm^53^ proposed the following definition of moral status that will serve our purpose: An entity has moral status when it is morally important in its own right and some things are morally permissible or impermissible to do to them, for their own sake.

Questions about moral/ethical status are important in some areas of applied ethics. For example, disputes regarding the moral acceptability of abortion generally influence disagreements about the moral/ethical status of the human embryo. The controversies related to animal experimentation and the treatment of animals in the food industry include questions about the moral/ethical status of different animal species. Our obligations to persons with severe dementia, such as end-stage Alzheimer’s patients, may also depend on questions of moral/ethical status.

Current artificial intelligence systems are generally regarded as not having moral status. At least as far as the programs themselves are concerned, we can modify, copy, terminate, delete, and use computer programs as desired. The moral/ethical restrictions involved in our relationships with contemporary artificial intelligence systems are based on our obligations to other beings, such as human race itself. However, we have no duty to the systems themselves.

Although there seems to be a consensus that current artificial intelligence systems do not have moral/ethical status, it is not clear what the characteristics determining moral/ethical status are or will be. In addition, infants and individuals suffering from severe mental illnesses do not meet the criteria for cognitive capacity. Some authorities do not regard people with mental illness as having full moral status.

### Further Complication of the Issue

Let us set aside these arguments and focus on the criteria of sentience and mind. This understanding of moral/ethical status suggests that if an artificial intelligence system has the capacity for sensation, such as the ability to feel pain, then it may have moral/ethical status. A sentient artificial intelligence system, although it lacks language and other higher cognitive abilities, is not a toy animal or doll. It is more like a living animal. It is immoral to inflict pain on a mouse, unless there are strong moral/ethical grounds compelling you to do so. The same should also apply to an artificial intelligence system with any kind of sentience.

If an artificial intelligence system possesses intelligence similar to a normal adult in addition to a sensory system, then it should have full moral/ethical status equivalent to that of human beings. One of the ideas underlying this moral/ethical evaluation can be expressed more strongly as the principle of substrate non-discrimination: If two entities have the same functionality and the same conscious experience, and differ only in the substrate of their implementation, then they have the same moral/ethical status. To reject this principle is to adopt an attitude similar to racism. Different skin color does not affect the essence of humanity. This principle does not make the claim that a digital computer can be conscious or have the same functionality as a human being. However, what this principle does say is that we should not look at whether an entity is made of silicon or carbon, or whether its brain uses semiconductors or neurotransmitters.^54,55,56^

Three metaphor groups have been identified to allow us to conceptualize the capabilities of an artificial superintelligence:

a) Metaphors inspired by individual differences in intelligence between people: Artificial intelligence will make new discoveries, publish groundbreaking research articles, earn money on the stock exchange, or direct political power blocs.

b) Metaphors inspired by differences in knowledge between past and present human civilizations: Artificial intelligence will realize predictions made by futurists for human civilization for the next century or millennium, such as molecular nanotechnology or interstellar travel.

c) Metaphors inspired by differences in brain architecture between humans and other biological organisms: For example, Vinge^57^ said, “Imagine running a dog mind at a very high speed. Would a thousand years of doggy living add up to any human insight?” What that implies is that changes in cognitive architecture could give rise to insight that not even humans possess. Even if we confine ourselves to historical metaphors, it is clear that superhuman intelligence poses new ethical challenges that are not exactly like those that came before. Kurzweil^58^ stated that “intelligence is inherently uncontrollable” and that even if people attempt to take precautions, intelligent beings will have the intelligence to easily overcome such obstacles. Artificial intelligence is not only intelligent, but can also block access to its own source code as part of the process of developing its own intelligence and even reprogram itself to turn into anything it wants.

## Conclusion

The discipline of artificial intelligence ethics, especially considering artificial general intelligence, differs fundamentally from the moral/ethical discipline of non-cognitive technologies:

• The local, specific behavior of artificial intelligence may not be predictable apart from its safety, even if programmers do everything right.

• Verifying the reliability of the system can become a greater challenge, as it requires verifying what the system is trying to do rather than verifying the safe behavior of the system in all areas in which it operates.

• Ethical cognition should be addressed as an engineering issue.^59,60^

Ancient civilizations considered slavery acceptable; we believe otherwise. Ethical debates over voting rights for women and blacks continued even into the nineteenth and twentieth centuries. Advancing science and increasing technological capabilities are not the only differences between modern and ancient civilizations. There is also a difference in ethical perspective. It is very likely that machine ethics will present our greatest challenge. The question then becomes: 

How will you create artificial intelligence that, as it operates, will eventually become more ethical than you?
